# How Can We Improve Disease Education in People with Gout?

**DOI:** 10.1007/s11926-018-0720-x

**Published:** 2018-03-08

**Authors:** Theodore R. Fields, Adena Batterman

**Affiliations:** 1 Division of Rheumatology, Hospital for Special Surgery, 535 East 70th St., Suite 848-West, New York, NY 10021 USA; 2 Weill Cornell College of Medicine, New York, NY USA; 3 Department of Social Work Programs, Hospital for Special Surgery, New York, NY USA

**Keywords:** Gout, Patient education, Self-management, Adherence, Crystal arthritis, Multi-disciplinary care

## Abstract

**Purpose of Review:**

Gout management is currently suboptimal despite excellent available therapy. Gout patient education has been shown to enhance medication adherence and self-management, but needs improvement. We explored the literature on gout patient education including gaps in gout patient knowledge; use of written materials; in-person individual and group sessions; education via nurses, pharmacists, or multi-disciplinary groups; and use of phone, web-based, mobile health app, and text messaging educational efforts.

**Recent Findings:**

Nurse-led interventions have shown significant improvement in reaching urate goals. Pharmacist-led programs have likewise succeeded, but to a lesser degree. A multi-disciplinary approach has shown feasibility. Needs-assessments, patient questionnaires, and psychosocial evaluations can enhance targeted education.

**Summary:**

An interactive and patient-centered approach can enhance gout educational interventions. Optimal programs will assess for and address educational needs related to knowledge gaps, health literacy, race, gender, socio-economic status, and level of social support.

## Introduction

The need for effective gout patient education is powerfully supported by the literature and medication adherence is acknowledged as a key goal in improving rheumatic disease health outcomes [1]. Fortunately, there is evidence that patients with gout can improve adherence with appropriate education [2].

Gout is common [3], has a significant negative impact on quality of life [4–6], and has an increased risk of mortality [7]. The economic burden of gout is high [8]. Current treatments for gout, if carefully prescribed and regularly taken, are effective in stopping gout flares and improving quality of life in the vast majority of patients [9–11]. All but one published gout treatment guidelines support the importance of a serum urate goal, generally < 6.0 mg/dl for those with non-tophaceous gout [12–14]. Despite these guidelines and the availability of highly effective therapies for reducing serum urate, the majority of gout patients, internationally, have poor medication adherence and fail to reach their serum urate treatment goal [15–19]. Even when appropriate prescribing and treatment practices are followed, prescriptions are often not taken as directed.

The 2017 British gout management guidelines strongly emphasized the importance of gout patient education, which should highlight key management points, such as causes and consequences of gout and hyperuricemia, lifestyle and diet, alcohol and obesity, and urate-lowering therapy (ULT) goals. These guidelines emphasize an individualized education plan, “considering co-morbidities and concurrent medications, illness perceptions and potential barriers to care” [12]. Inaccurate popular beliefs and myths about gout held by patients and physicians present barriers to appropriate patient education and treatment and need to be addressed and dispelled [20]. All of these findings indicate that quality improvement approaches in gout care “clearly start with provider and patient education” [21].

Gout patient education has been suboptimal in outpatient settings [18, 22••]. For example, patients with gout tend to be unaware of their urate goal, which is the cornerstone of long-term management [23]. In the hospital setting, patients are likely to leave hospital without a urate-lowering plan [24].

Patients with gout are primarily managed by primary care physicians (PCP’s), who are time-challenged and need to be kept updated on current optimal gout management strategies, especially in patients with multiple comorbidities. Rheumatologists, therefore, need to educate their primary care colleagues and other providers involved in the care of gout patients.

Patient self-management is a key part of chronic disease care [25], and structured programs to enhance self-management efforts have been shown effective in arthritis [26] and in other chronic conditions [27]. The need to include families and lay caregivers in patient education to improve outcomes has been emphasized in chronic diseases, and also in gout [28, 29].

Recently, several gout-specific patient education interventions have been studied, including nurse- and pharmacist-led programs. A multi-disciplinary team approach to patient education has been increasingly studied [30].

The literature supports the need for a patient-centered approach to education for rheumatic diseases, including gout, which addresses the unique psychoeducational needs of individual patients [31–33]. This review will discuss the available literature on disease-specific education in people with gout and suggest directions for future research.

## What Do Gout Patients Need to Know? What Are Their Knowledge Gaps?

A recent EULAR panel reviewed the literature regarding patient education interventions for people with inflammatory arthritis. The review underscored the need for an interactive, ongoing dialogue to address disease-specific information, and also the unique emotional and informational needs of each patient [34].

Gout patient disease- and treatment-related knowledge gaps have been well explored in the literature. A ten-item patient questionnaire assessing for gout disease and treatment knowledge reported a mean number of correct responses of 6.15, with frequent incorrect responses related to use of urate-lowering drugs, optimal goal urate levels and appropriate duration of ULT [35]. Similar gaps were found in other studies [36, 37]. Knowledge gaps related to lifestyle modifications, including alcohol intake and dietary triggers for gout attacks, are also widely reported [36, 38].

A 26-item needs assessment was administered to 100 rheumatologist-treated gout patients, to obtain information about knowledge gaps, perceived self-efficacy, and patient-identified psychoeducational needs and preferences [39]. This study found that patients had difficulty understanding bridge therapy, the use of prophylaxis for gout flares during the early phase of urate-lowering therapy. They were often unaware of optimal treatment goals (e.g., “no further flares”). For example, more than 30% of study participants who had > 3 flares within the past 6 months felt that their gout was “under control.” Respondents expressed the desire for additional information about managing medication side effects and dietary management, specifically from a nutritionist. This prompted inclusion of this content and a nutritionist as speaker for an upcoming community gout symposium.

Educational gaps exist for caregivers as well as patients. An online survey which looked at the impact of gout on quality of life of a large number of gout patient caregivers and patients identified these gaps (https://creakyjoints.org/goutsurvey/). Gaps included limited understanding of the role of diet and genetics in gout, and poor knowledge of the target urate goal. These findings indicate that caregiver education might enhance patient adherence and quality of life and is consistent with a recent proposed emphasis on patient- and family-centered medical education [40].

Persistent myths, such as the lack of a need for long-term ULT, have been analyzed [36] and patient education discussions should include clarifying misconceptions. These issues, as well as other barriers to optimal care unique to each patient, should be explored, not only in initial consultations but as part of an ongoing dialogue. Also, tools such as gout knowledge questionnaires can be used as initial screens to identify misconceptions and other knowledge gaps and can be useful in guiding patient-provider discussions. Several such questionnaires have been published [35–37]. Gout patients often lack knowledge of the “causes and consequences” of gout, with little appreciation that poor medication adherence leads to recurrent gout flares. Many associate gout with negative stereotypes [28] and with images portrayed in Victorian cartoons, and see their disease as self-inflicted [41]. With a lack of understanding of the genetic and metabolic aspects underlying their gout, patients might see themselves as “at fault.” Patients have expressed feeling stigmatized by healthcare providers, who they believe perceive their gout as related to alcohol and dietary excess. These issues can affect patients’ willingness to seek treatment or engage in open dialogue about their symptoms with providers [42, 43].

## Patient-Centered Education: Incorporating the Patient Perspective

Research which underscores benefits of incorporating the patient’s perspective in research and program initiatives is well established [44, 45]. These studies provide insight into what symptoms, experiences, and treatment outcomes have meaning for and relevance to patients and can be used to inform content for educational interventions. Interventions should ideally emphasize issues which motivate patients to adhere to treatment based on their priorities and identify barriers to self-management. In rheumatoid arthritis (RA) literature, one well-designed study reported on an educational needs assessment, which asked patients to prioritize their educational needs via a questionnaire. Results were used to develop a needs-based education program, which improved participants’ self-efficacy and health status [33]. Though this assessment was studied in RA patients, a similar approach might be considered to assess for patient-identified needs for individualized gout education.

Several recent qualitative studies have focused on gout patients experiences of gout and gout-related education. Khanna et al. conducted focus groups of gout patients and reported that they felt they lacked sufficient knowledge of treatment options, the progressive nature of gout, and felt that practitioners did not spend enough time with them on education [46]. Other studies report similar themes with additional disease and treatment education concerns, such as the need for warnings about increased risk of gout flares upon initiation of ULT, and lack of information about diet. Of note, patients expressed that they would have started ULT sooner, had they better understood the rationale for treatment [36, 42]. Another qualitative study looked at patient-identified core gout-related issues impacting patients’ quality of life, including pain, sleep, and social activity [47].

### Race and Gender: Impact on Gout Educational Needs

Race and gender disparities in gout management have been explored. Krishnan looked at sociodemographic variables associated with allopurinol prescription during outpatient visits for patients with gout, and found that African Americans were less likely to receive allopurinol than Caucasians, and an even lower proportion of Asian Americans received allopurinol prescriptions [48]. African Americans have a higher prevalence of gout risk factors but, after accounting for these risk factors, have a significantly lower risk for both gout and hyperuricemia compared with Caucasians [49]. Racial differences in patient-reported quality of life, and barriers and facilitators to adherence have also been studied. For example, qualitative studies of African-American patients with gout found a number of barriers to urate-lowering therapy adherence, including cost and concerns about side effects of medication, patient preference for alternative medicines, concerns about dietary restrictions, and doubts regarding effectiveness of medication [49–51]. Facilitators to adherence included understanding of role of ULT in flare prevention, pain prevention, benefit of less dietary restrictions, and trust in physician [50, 51].

A large body of literature discusses the influence of culture, race, and ethnicity on patient experience of medical encounters and systems. All of these have impact on patient/provider rapport, trust, and communication, including how patients ask questions, their comfort level in raising concerns about treatment and sharing cultural perspectives about illness [52]. Discordance in race/ethnicity between provider and patient can also present challenges in patient education related to language barriers when communicating complex disease and treatment information, and, discussing gout patients’ culture-related differences in illness perceptions, self-management, and treatment [53].

Differences in impact of gout on gender have also been identified. In one study, women were especially concerned with increased dependency and joint deformities as a result of progressing gout [38, 51]. Another study found women participants to have difficulty accepting a diagnosis of gout and finding information relevant to them, influenced by common beliefs and stereotypes that gout is a condition typically affecting men. Women in this study were likely to feel that gout had a major impact on their identity, roles, mood, and relationships and found it helpful to reach out to other women for support online [38]. Another recent study found that men with RA may be less open to support group sessions than women and suggest that gender-related concerns, impact of illness, and preferences for support should be considered when developing strategies and interventions for education and support [54].

## Gout Patient Educational Interventions: Multiple Modalities

### Written, Online, and Mobile Health Materials

A review of patient information about gout, written and online, found that over half were written at a “highly complex level” and that some key areas were inadequately covered, such as comorbidity-related risks and the importance of continuing urate-lowering therapy during attacks [55]. This review also found essential self-management information such as dietary guidelines and serum urate target levels were inconsistently presented within resources. A 2013 study reviewed ten gout patient information resources and found the median readability grade level was 8.5, and approximately one third of these resources were found to be above the average reading level of rheumatology out-patients. Sixty percent of resources lacked essential information about gout flare prophylaxis during initiation of urate-lowering therapy and treating serum urate to target [56]. A recent article emphasized the need for specific language about gout that will best help patients (and providers) understand the concepts being taught [57].

Mobile applications for gout have been developed and show promise for future interventions which use interactivity to tailor information to individual self-management needs [58]. One review [59] examined 57 English-language gout apps, 6 of which allowed patients to monitor their urate levels and gout flares along with educational materials. One included all three of these elements, but the majority did not function within the app and required users to print out and complete materials. A review of digital health programs to improve adherence in multiple rheumatic diseases, including gout, emphasized the need for tailored, patient-centric interventions that can focus on individual patterns of non-adherence [32]. Electronic reminders to improve adherence to chronic medications have also been reviewed. In a study of use of text (SMS) messaging, electronic reminder devices, and pagers to improve medication adherence in several medical conditions, improvement was found, especially with text messaging [60]. The optimal online program may be a combination of interactive education, documentation in a format easily shared with a health care provider and an alert system, a combination not yet available.

A number of online tools are available for gout patient education, including the Gout & Uric Acid Education Society, http://gouteducation.org/, the American College of Rheumatology (https://www.rheumatology.org/I-Am-A/Patient-Caregiver/Diseases-Conditions/Gout) and Hospital for Special Surgery (https://www.hss.edu/conditions_gout-risk-factors-diagnosis-treatment.asp). Further study is needed to assess the impact of these tools and their appropriate role in education and management.

## Individual and Group Patient Education Interventions

Reviews of the effects of educational efforts on adherence have shown variable results, with best results found in interventions tailored to patients and delivered by a healthcare provider [29, 61]. Reviews have shown progress in overcoming barriers to gout patient education and self-management, but with more gains yet to be made [62].

### Individual Interventions

A pharmacist-coordinated program reported a greater number of intervention patients reaching their goal urate of < 6.0 mg/dL, but this reflected only 13 (35%) of the intervention patients vs. 5 (13%) of control group patients [63]. Another group looked at pharmacist-directed phone-based gout patient education and, in a recent abstract, reported that the intervention patients reached their serum urate goal more frequently than controls, but only 31.3% of 631 intervention patients vs. 20.6% of 782 control patients reached urate < 6.0 mg/dL [64].

An observational study of a nurse-coordinated intervention showed that 92% of the 106 participants reached urate < 6.0 mg/dL at 12 months [65••]. The intervention involved a clinical assessment with a rheumatologist which included gout-related education, an individualized tailored plan addressing modifiable risks and ULT information. Ongoing follow-up with a nurse included counseling about chronic disease progression, lifestyle self-management advice, and availability for questions as needed. Five years post-intervention, persistence on ULT was 90.7%, per patient-report via a questionnaire [66]. In another study, positive effects of nurse-provided drug information have also included increased patient sense of autonomy, knowledge, and trust in their caregiver [67].

Given the strong support in the literature for using a team approach to augment physician-provided gout education, our team recently reported on a pilot study of a gout education and management program which included a multi-disciplinary team of pharmacists, nurses, and a social worker [68]. The study featured a nurse-led patient education initiative and used a standardized curriculum covering gout causes, treatment, medications, urate goals, and dietary recommendations. Administration of a gout knowledge questionnaire prior to teaching was used to identify targeted content for individual counseling with a specially trained nurse. Follow-up phone calls were made monthly by pharmacists, to encourage adherence and respond to medication questions. The study supported the feasibility and acceptability of implementing a team approach to gout patient education in a busy rheumatology academic practice and suggested future controlled studies of such programs.

### Group Interventions

Group interventions aimed at enhancing education and self-management strategies for gout patients have not been widely reported in the literature, but have been discussed in other rheumatologic conditions with mixed results. These can, perhaps, provide guidance in creating similar gout-specific patient interventions. For example, the Arthritis Self-Management Program (ASMP) is a well-studied, structured intervention which focuses on self-management strategies for arthritis patients, and has reported improved pain, increased disease-related knowledge, and recommended health behaviors compared to a control group patients [26]. The program has also been successfully duplicated with positive results for Spanish-speaking patients, including improved health behaviors, health status, and self-efficacy [69].

Other similar group interventions have reported improvements in depression, social and role limitations, and patient-provider communication [27]. A nurse-led patient education group intervention with a follow-up individual session was studied in patients with inflammatory arthritis. This was a 6-week program which focused on problem-solving, self-management skills, goal setting, and treatment options. The last 26 patients enrolled in this randomized, controlled study were interviewed before and 2 months after completion of the intervention. These patients (15 in the intervention group and 11 in the control group) in this qualitative study were questioned about their perceived coping with their arthritis. Patients in the educational intervention group, but not in the control group, reported strengthened confidence in managing pharmacological treatment, better coping with illness fluctuations, and positive changes in health behaviors (including improved medication adherence and dietary management). Intervention group participants attributed these improvements, in part, to the group modality; they identified the specific benefit of participating in a forum to discuss issues related to illness management and coping with peers with shared experiences [70]. While these results provide support for the value of a group modality in patient education for inflammatory arthritis, potential limitations should be kept in mind in interpreting the results. As the authors acknowledge, selection bias may have occurred with patients at initial entry into the randomized trial. Patients who felt they were coping well may have declined participation since they did not feel a need for education. Alternatively, patients with poorer coping skills may have been less able or likely to participate in an intervention which required multiple sessions and interviews. In another study which reported on patient perceptions of a cognitive behavioral therapy (CBT) group intervention for managing fatigue in RA, patients reported enhanced self-management efforts [71]. Although results on the efficacy of group patient education interventions have yielded mixed results, there is sufficient evidence that suggests that further research should be conducted to determine which models might be used effectively in gout patient education and which patients would benefit.

## Additional Considerations

### Health Literacy

There is strong evidence to suggest that many patients do not understand disease-related information provided in written and verbal form in rheumatology medical encounters [72]. Many patients may leave a rheumatologist’s office with poor recall, retaining only a small portion of the information discussed at their appointments [73]. Also, patients who are overwhelmed, anxious, or depressed have difficulty retaining and processing health-related information. Providers cannot assume patients’ health literacy competency based on education level alone.

Simple brief validated screens, such as the Single Item Literacy Screen (SILS), can be used as a preliminary evaluation, to determine if a patient has literacy and language barriers or other barriers which might impact their ability to read health-related materials. Engaging all patients in bi-directional teaching, including “teach-back” techniques, can be effective in determining whether the patient has understood information, can provide an opportunity to clarify key issues, and can improve disease-related knowledge, adherence, and self-efficacy [74]. A toolkit for rheumatology healthcare providers to help address issues of health literacy can be found at http://www.med.unc.edu/tarc/files/HLUPTRheum.pdf.

## Opportunities and Timing of Education

The acuity and severity of gout flares can be powerful motivators to take preventive measures and can present a unique opportunity to discuss long-term management plans. One study reviewed charts of hospitalized patients with gout flares and found that only 36.1% had ULT initiation or dose alteration in the hospital or recommendations made to their general practitioners. This percentage increased to 82.1% if the patient was seen by the rheumatology service [24]. Similar critical patient education opportunities may occur when patients are seen for gout flare in emergency rooms or urgent care centers or by podiatrists. Both providers and patients need to see these episodes as opportunities to improve long-term prevention strategies.

Repeated educational messages (spaced learning) have been shown to be an effective strategy for memories that “stick” [75] This implies the benefit to having periodic “refresher” sessions with gout patients to remind them of the rationale for their long-term use of ULT and other key self-management points. Feedback, where patients receive individualized information based on their knowledge deficits or specific questions, has been shown to improve adherence to medications [76]. This supports potential benefit of interactive knowledge questionnaires with feedback on responses, and online programs which provide education informed by individual details entered by the patient.

## Conclusions and Future Options

**Fig. 1:**
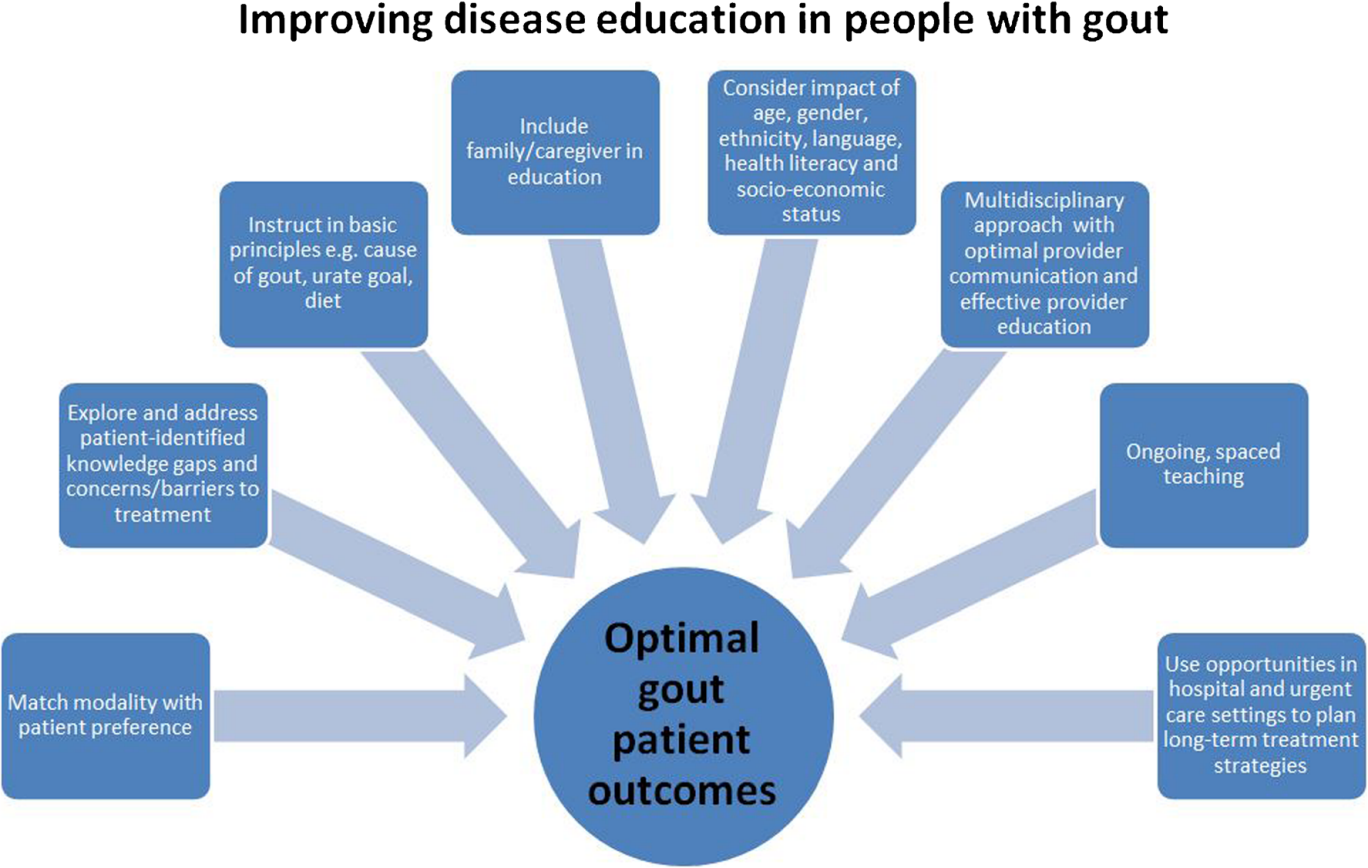
Factors to consider in a patient-centered interactive approach to gout patient education

The rewards of gout patient education in terms of improved outcomes seem clear, but much work still needs to be done. Review of the literature and our experience suggests that future optimal education for patients with gout requires a multi-faceted approach (see Fig. 1). Multiple types of health care professionals have the potential to offer excellent gout patient education, either individually or in labor-dividing teams. Approaches must incorporate patient-identified needs and also ensure that specific knowledge gaps and best practice clinical management guidelines are addressed. Written or online questionnaires can help identify such gaps and have the potential to help create targeted individualized patient education. Key concepts highlighted in the literature include genetic and metabolic causes of gout, the risk of progressive damage with inadequate treatment, goal serum urate levels and why they are monitored, rationale for short- and long-term management, impact of comorbidities, and dietary management. Focus on these issues reflects partnership between patient and provider, where both are working toward a shared concrete goal. As a team, providers must assess for possible barriers to treatment, such as ability to pay for medications and health literacy issues. Patients need to be screened for health literacy, and providers can ensure that information is well understood, using strategies such as teach-back methods [77] and reading level-appropriate materials [72]. Caregivers and family, as well as gout patients, need to be educated. This is consistent with the developing goal of patient- and family-centered medical education [40].

Also critical to improving gout patient education is addressing gout health care provider education. Efforts to improve gout management knowledge of primary care providers and other providers who are involved in gout patient care is critical to ensuring gout patients get the essential information they need to support them in their self-management efforts. The importance of close monitoring of serum urate levels and optimal treatment goals are key points to emphasize. Further, primary care physicians and other providers should be encouraged to consult with a rheumatologist when, even after multiple adjustments to treatment are made, a patient cannot reach urate goals, or continues to experience refractory gout flares. The future of gout patient education must also include the many settings where critical educational opportunities arise. Patients with gout flares in the hospital [24], emergency rooms, and podiatric offices should be educated about long-term prevention as well as the immediate issue of gout flare management.

Optimal disease education and self-management tools for gout patients must consider patient preferences and modalities most effective for them. Online programs may be appropriate for some people, and support and education groups or individualized patient counseling for others. Some will prefer text messages reminders; others may prefer email alerts or phone calls. Different approaches may be needed based on age, educational level, health literacy, gender, ethnicity, language, and other factors which affect ability to access programs. Further research is necessary to look at which patients would benefit from interventions, and identify barriers to access.

Self-management education programs have demonstrated positive impact on self-efficacy, knowledge, recommended behaviors, and pain [26]. Despite this, participation among US adults remains persistently low (especially in patients with less formal education), though attendance is increased when treating providers make direct referrals to these programs [77]. These findings suggest a need to target promotion of appropriate programs and their benefits to physicians as well as to patients.

Given the time constraints of increasingly brief medical encounters, it is challenging to adequately address the complex information required for optimal self-management and then further, to ensure that this information is well understood, reinforced, and retained, with patient concerns and questions appropriately explored. Nurses, physician assistants, nurse practitioners, pharmacists, and nutritionists with relevant specific training in gout management and treatment all play an essential patient counseling role. Social workers should be a part of this multi-disciplinary approach, as they are trained to assess and explore issues around coping with chronic illness and treatment, including the financial, social and cultural factors which can impact patients’ understanding, decisions, and adherence in a profound way. Effective communication between multiple providers around patient care is also essential to ensure that consistent messages are given to patients, and treatment plans are understood by all clinicians involved [31].

Culture, race, and ethnicity influence patients’ trust in asking questions, expressing concerns about management and treatment, and sharing cultural perspectives about illness beliefs and treatments. Providers must initiate and encourage bi-directional discussions about patients’ perspective and encourage questions so that education, treatment, and self-management are relevant to the unique circumstances of each patient. Explicitly asking about and incorporating these issues is an important ingredient in building patient-provider trust and rapport. This is a foundational element of patient-focused education and care and has impact on adherence and health outcomes [78].

In spite of effective available treatments for gout, patients continue to have suboptimal outcomes. Ongoing research is needed to identify and evaluate initiatives which can successfully and cost-effectively support patients in their efforts at self-management, using modalities which are relevant and tailored to individual patient needs. Effective gout patient education is a powerful tool in enhancing self-management strategies and, ultimately, can have a significant impact on gout treatment outcomes and patient quality of life.
